# Adapting gas fermenting bacteria for light-driven domino valorization of CO_2_†

**DOI:** 10.1039/d5sc00764j

**Published:** 2025-05-12

**Authors:** Lin Su, Santiago Rodríguez-Jiménez, Marion I. M. Short, Erwin Reisner

**Affiliations:** a Yusuf Hamied Department of Chemistry, University of Cambridge Cambridge UK reisner@ch.cam.ac.uk

## Abstract

The solar-driven valorization of CO_2_ to fuels and chemicals provides an exciting opportunity to develop a circular chemical industry, but the controlled production of multicarbon organics remains a major challenge. Here, we present an abiotic–biotic domino strategy that integrates a photocatalytic CO_2_-to-syngas conversion system with evolved syngas-fermenting bacteria to enable the upcycling of CO_2_ into valuable C_2_ products, including acetate and ethanol. To optimize microbial syngas fermentation through an accessible and chemist-friendly platform, we employ adaptive laboratory evolution (ALE) of *Clostridium ljungdahlii* (*Cl*). The adapted strain, *Cl*_adapt_, exhibits a 2.5-fold increase in growth rate and a 120-fold enhancement in C_2_ production compared to the wild type (*Cl*_wt_). Isotopic labeling confirmed *Cl*_adapt_'s high conversion efficiency, yielding 6 : 1 and 9 : 1 ratios of ^13^C : ^12^C in acetate and ethanol, respectively. Whole genome sequencing revealed mutations in *Cl*_adapt_, offering initial clues to its enhanced metabolism. A scaled-up semiconductor-molecule hybrid photocatalyst, TiO_2_|phosphonated Co(terpyridine)_2_, was employed to generate sufficient syngas (CO/H_2_ ratio: ∼30 : 70 with 1.3 mmol of CO after 6 days) for *Cl*_adapt_ to demonstrate photocatalytic CO_2_ → syngas → C_2_ conversion (yielding 0.46 ± 0.07 mM, or 3.2 μmol, of acetate). This study offers a streamlined approach to improving syngas fermentation in *Cl*, insights into microbial adaptability, and an ALE-guided pathway for solar-powered CO_2_ upcycling using an inorganic-microbial domino strategy.

## Abstract 2

The solar-driven valorization of CO_2_ to fuels and chemicals provides an exciting opportunity to develop a circular chemical industry, but the controlled production of multicarbon organics remains a major challenge.

## Graphical abstract

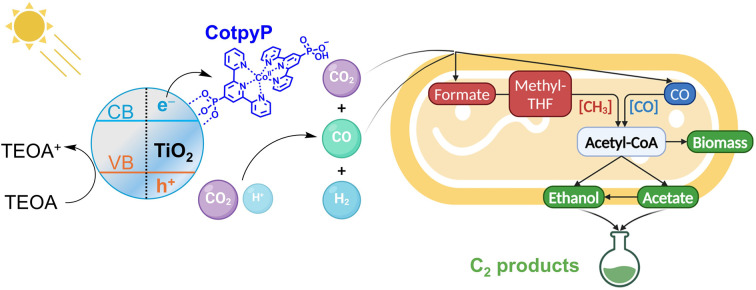

## Introduction

Semi-biological photosynthesis merges synthetic and microbial approaches to produce sustainable fuels using sunlight, particularly excelling in selective multicarbon product formation *via* the metabolic pathways available through biocatalysis.^1,2^ Several recent reports demonstrate the promise and feasibility of this emerging approach: A hybrid solar water splitting–biosynthetic system uses green H_2_ as intermediate to fix CO_2_ and efficiently produce biomass and fuels using *Cupriavidus necator*.^3^ Coupling a synthetic photocatalyst with *Shewanella oneidensis* resulted in selective hydrogenation of C=C and C=O bonds.^4^ The photosensitization of *Moorella thermoacetica* with extracellular CdS or intracellular gold nanoclusters established a pathway for bacterial CO_2_ utilization, integrating microbial systems with nanomaterials to enhance photosynthetic efficiency.^5,6^

**Fig. 1:**
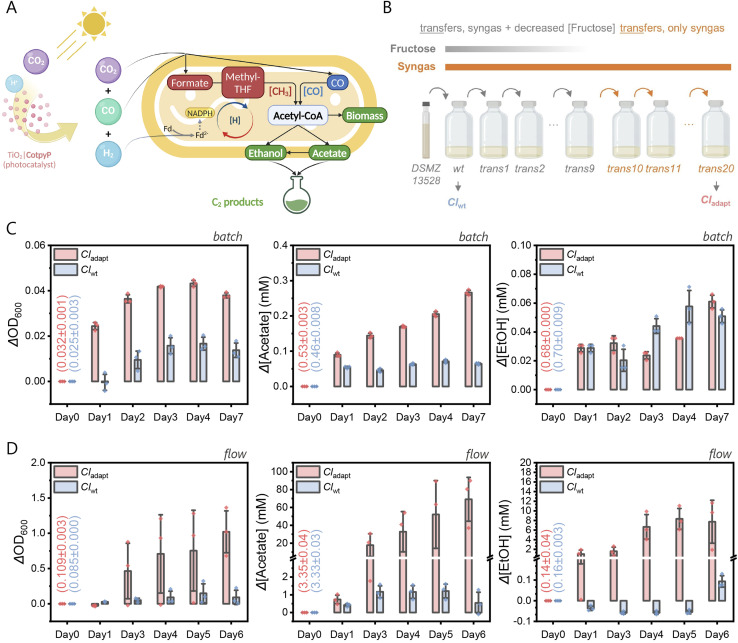
Adaptive laboratory evolution (ALE) of *C. ljungdahlii*. (A) Schematic illustration of the syngas-fermenting metabolic pathways in *C. ljungdahlii* (*Cl*), including integration with photocatalytic syngas production. [CH_3_] (red color), methyl branch; [CO] (blue color), carbonyl branch. (B) Schematic summary of the ALE process. The wild-type strain (*Cl*_wt_) was adapted to a syngas environment through gradual fructose reduction and removal over 20 transfers, resulting in the adapted strain (*Cl*_adapt_). (C) Comparative analysis of growth and C_2_ product generation (acetate and ethanol) between *Cl*_adapt_ and *Cl*_wt_ under batch gas purging conditions (syngas purging for 30 min daily, 112 mL headspace). (D) Similar comparative analysis under continuous gas flow conditions (10 mL syngas per min). Growth conditions: ATCC Medium 1754 (pH 5.9), 37 °C with 150 rpm mixing. Numbers in brackets indicate the values at the start of the experiment.

Gas fermenting acetogenic bacteria, particularly *Clostridium* species, have emerged as versatile platforms to produce biofuels and biochemicals from syngas, a mixture of H_2_, CO and CO_2_.^7–9^ These *Clostridium* strains utilize the Wood–Ljungdahl pathway, which consists of two branches to produce the C_2_ compounds (Fig. 1A): the methyl branch, reducing CO_2_ to formate and further to a methyl group, and the carbonyl branch, which forms an acetyl group from CO. *Clostridium ljungdahlii* (*Cl*) has shown promise in fermenting these gases into valuable products, underscoring the economic viability of this approach in biotechnology. For instance, a carbon-negative fermentation process was established using engineered and closely related *Clostridium autoethanogenum* to convert waste gas feedstocks into acetone and isopropanol with high efficiency.^10^ An Ag-catalyst based gas diffusion electrolyzer for CO_2_-to-syngas conversion was coupled with syngas-fermenting *C. autoethanogenum* and *C. kluyveri*, producing butanol and hexanol with high selectivity and therefore offers a sustainable pathway for industrial chemical production from CO_2_ and water using renewable energy.^11^

To further harness and optimize the metabolic capabilities of these microorganisms, adaptive laboratory evolution (ALE) emerges as a powerful tool to select and enhance beneficial traits without the need for genetic engineering. The principles and applications of ALE have demonstrated its simplicity and efficacy in tailoring microbial phenotypes for improved industrial performance.^12,13^ Examples of successful ALE applications illustrate the transferability, flexibility and transformative potential of ALE in metabolic engineering and synthetic biology.^14–19^ For example, ALE of *Sporomusa ovata* integrated with light-harvesting silicon nanowires led to a 2.4-fold increase in CO_2_-reducing current density, enhancing bioelectrochemical CO_2_ reduction.^16^ Similarly, *C. autoethanogenum* strains developed through ALE showed superior growth and product profiles in continuous bioreactor cultures.^19^ ALE using CO_2_ and H_2_, with other *C. autoethanogenum* lineages exposed to 2% CO, has also significantly enhanced growth rates and ethanol production, revealing extensive proteome and metabolome changes that highlight new targets for metabolic engineering.^20^

Here, we explore the synergistic potential of ALE-derived gas-fermenting bacteria and photocatalysis for the overall conversion of CO_2_ to acetate and ethanol (Fig. 1A). An adapted strain of *Cl* was developed and shows improved syngas-to-acetate conversion. The syngas for fermentation by *Cl* was subsequently sourced from a synthetic CO_2_-to-syngas photocatalyst to demonstrate an overall CO_2_ → syngas → acetate domino-reaction. We therefore present a novel approach for solar energy storage in multicarbon chemicals and biomass that harnesses the symbiotic strength of synthetic and biological catalysts.

## Experimental Section

### Materials and chemicals

Deuterium oxide (D_2_O) solution (99.9 atom% D) containing 0.05 wt. or 0.75 wt% 3-(trimethylsilyl)propionic-2,2,3,3*-d*_4_ acid, sodium salt, triethanolamine (TEOA, ≥99.0%) and glycerol (for molecular biology, ≥99.0%) as well as all chemicals used for bacterial growth and the isotopically labelled gases ^13^CO_2_ and ^13^CO were purchased from Sigma-Aldrich. TiO_2_ powder P25 (10–30 nm diameter; 50 m^2^ g^−1^) was obtained from Evonik. [Co(2,2′:6′,2′′-terpyridine-4′-phosphonic acid)_2_](BF_4_)_2_ (denoted as CotpyP) was synthesized and characterized according to reported procedures.^21^ Reaction gases CO_2_ with 2% CH_4_ as internal gas chromatography standard, and synthesis gas (syngas) cylinders (25% CO, 10% H_2_, 65% CO_2_) were purchased from Brin's Oxygen Company (BOC).

### Bacteria growth, syngas adaptation, storage and handling

*Clostridium ljungdahlii* (*Cl*) DSMZ 13528 was purchased from The Leibniz Institute DSMZ (German Collection of Microorganisms and Cell Cultures GmbH). Cultures were initially grown in ATCC Medium 1754, which contained essential nutrients and vitamins, supplemented with 5.00 g L^−1^ fructose as a carbon source to establish initial growth conditions conducive to syngas adaptation. The specific setups for feeding syngas (Fig. S1†), including batch and continuous flow modes, were designed to mimic industrial conditions and evaluate the strains' performance under varying operational parameters.

The adaptation to syngas was conducted in a two-stage process. In the first stage, freshly cultured wild-type *Cl* (*Cl*_wt_) cells were acclimatized to a syngas environment by gradually decreasing the fructose concentration in the ATCC Medium 1754 over nine consecutive transfers, each lasting 72 hours. This gradual reduction aimed to incrementally expose the microbial population to syngas with less dependency on the fructose, thereby selecting for cells with enhanced syngas utilization capabilities. The syngas (25% CO, 10% H_2_, 65% CO_2_) roughly mimics the syngas ratios obtained in typical industrial processes, such as biomass gasification, and state-of-the-art electrolysis systems.^22,23^ In the second stage, cultures were exclusively grown on syngas without any fructose supplementation for an additional 11 transfers. This stringent selection pressure ensured the enrichment of syngas-adapted phenotypes capable of sustained growth and metabolism solely on syngas.

Post-adaptation, the adapted *Cl* culture, designated as *Cl*_adapt_ (internal strain registration name as RLM034 for strain request), and the wild-type control, *Cl*_wt_ (internal strain registration name as RLM028 for strain request), were stored for long-term preservation. Cultures were mixed with an equal volume of sterile 40% glycerol solution (in ddH_2_O) to achieve a final concentration of 20% glycerol and stored at −80 °C. This method ensured the viability and genetic stability of the microbial samples for future analyses and experiments.

During bacterial cultivation and transfers, the medium bottles were continuously purged with N_2_ to minimize O_2_ exposure. Additionally, when taking optical density (OD) and nuclear magnetic resonance (NMR) samples, the medium bottles remained under syngas purging, further preventing oxygen intrusion.

### Optical density, product quantification, and isotopic labelling

The optical density was monitored at 600 nm (OD_600_) to assess cell growth and adaptation progress at each transfer stage using an ultraviolet-visible (UV-vis) spectrophotometer (Agilent Cary 60).

Gaseous H_2_ and CO in the headspace were analyzed by gas chromatography using a Shimadzu Tracera GC-2010 Plus with a barrier discharge ionization detector, equipped with a ShinCarbon micro–ST Column (0.53 mm diameter) kept at 40 °C using Helium carrier gas. Typically, 20, 50 or 100 μL of headspace gas were injected using a gas-tight syringe (Hamilton). The response factors for the gases were determined by calibration with known amounts of H_2_ and CO.

Fructose consumption and the production of formate, acetate and other C_2_ compounds, indicative of the metabolic activity and efficiency of syngas conversion by the adapted and wild-type strains, were quantified by quantitative ^1^H nuclear magnetic resonance (qNMR) spectroscopy using a Bruker 400 MHz Neo Prodigy Spectrometry. The chemical shift (*δ*) of the ^1^H NMR spectrum is referenced against the internal standard 3-(trimethylsilyl)propionic-2,2,3,3-*d*_4_ acid, sodium salt (0.05 wt% or 0.75 wt% in D_2_O). To prepare a typical NMR solution, 570 μL of a filtered aliquot (0.22 μm syringe filter) from the bacteria culture was combined with 30 μL of the internal standard solution in an NMR tube.

For isotopic labelling experiments, a sealed bottle containing a bacterial culture of *Cl*_adapt_ in ATCC Medium 1754 (modified) was purged with ^13^C-labelled syngas gas (65% ^13^CO_2_/25% ^13^CO/10% H_2_) using mass flow controllers (Alicat Scientific). The ATCC Medium 1754 was modified by changing the NaH^12^CO_3_ into NaH^13^CO_3_, for the purpose of ^13^C labelling. When indicated, 30 mM of ^13^C-formate was also added into the medium. The culture was kept in a shaker-incubator with a constant temperature of 37 °C and shaking (150 rpm) for 12 days. Subsequently, the products in the media were characterized by ^1^H NMR spectroscopy as described above.

### Calculation on growth rate and doubling time

The growth rate is highest and most consistent during the exponential phase. The growth rate (*μ*) can be calculated using the formula:
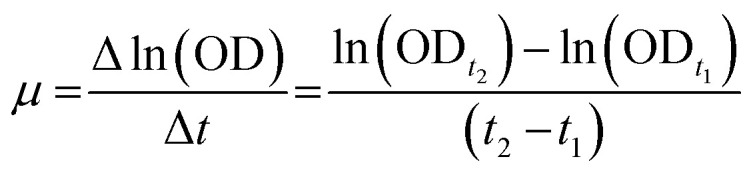
where Δln(OD) is the change in the natural logarithm of OD, and Δ*t* is the change in time (difference between time point 1 (*t*_1_) and 2 (*t*_2_)).

A python script was developed to analyze bacterial growth from optical density (OD) measurements over time. It automatically detects the exponential growth phase within a given time range, calculates the growth rate and doubling time, and plots the growth curve with the exponential fit. Source code is available on https://github.com/su2lin/microbiology.

### Sequencing

Whole Genome Sequencing (WGS) of the adapted strain (*Cl*_adapt_) and its wild-type counterpart (*Cl*_wt_) was performed to identify genomic variations associated with enhanced syngas fermentation capabilities. Sequencing and analysis were conducted by GENEWIZ. For each strain, a single cell pellet sample was prepared by centrifuging 13 mL of liquid culture to remove the culture medium. The resulting pellets were resuspended in DNA/RNA Shield reagent (Zymo Research Corporation), flash-frozen in liquid nitrogen for stability, and shipped on dry ice to ensure sample integrity during transit.

For WGS data processing, raw sequencing reads were initially assessed for quality using FastQC. Poor quality reads and bases were then filtered and trimmed using Trimmomatic. The quality-filtered reads were mapped to the *C. ljungdahlii* reference genome using the Burrows Wheeler Aligner (BWA), which is designed for mapping low-divergent sequences against large reference genomes. The resulting SAM files were converted to BAM format and indexed using samtools to facilitate visualization of the alignments. Finally, single nucleotide variants (SNVs) were detected using bcftools. This comprehensive bioinformatics pipeline—encompassing quality control, trimming, mapping, indexing, and SNV detection—ensured accurate identification of genomic variations between *Cl*_adapt_ and *Cl*_wt_.

### Photocatalytic syngas production

In a typical experiment, an aqueous suspension containing TiO_2_ (P25, 365 mg) and CotpyP (20 μmol g_TiO_2__^−1^) in 0.22 L of 0.1 M TEOA solution (pH ≈ 7) was added to a scaled-up photoreactor^24^ (total volume = 1.52 L, Fig. S2†). Then, the photocatalytic suspension was purged under stirring (400 rpm) with CO_2_, containing 2% CH_4_ as gas chromatography internal standard, for 45 min. The stirred photoreactor was subsequently irradiated from the top with a solar light simulator (G2V Sunbrick LED Solar Simulator) with *λ* = 366 nm to 423 nm (20.4 mW cm^−2^) for 24 h at ambient temperature. The irradiation provided by the solar light simulator for *λ* = 366–423 nm is consistent with AM1.5 G irradiation, but wavelengths *λ* > 423 nm were removed to avoid unnecessary heating of the photoreactor.

The photocatalytic process was monitored by analyzing the headspace (volume = 1.30 L) after completion (24 h, batch mode experiment) or every 24 h (flow mode experiment) to monitor H_2_ and CO formation using a gas chromatograph (see above). For the batch mode experiments, after 24 h of irradiation, the photoreactor was connected for 6 days to a bioreactor containing ≈13 mL of ATCC Medium 1754 with the adapted bacterial strain. For the flow mode experiments, the photoreactor was connected before solar light irradiation to three bioreactors and during irradiation, CO_2_ was flowed using a mass flow controller (Alicat) at a constant flow rate 30 mL min^−1^ and every 24 h, a fresh batch of 20 μmol CotpyP g_TiO_2__^−1^ was added into the photoreactor. Photocatalysis batch and flow experiments were performed in triplicates.

### Statistical analysis

The mean (*x̄*) with its standard deviation (*σ*_*x̄*_) expressed as *x̄* ± *σ*_*x̄*_ from *n* (*n* ≥ 3) independent experiments (*x*_*i*_). The standard errors were calculated using OriginPro 2024 (OriginLab).
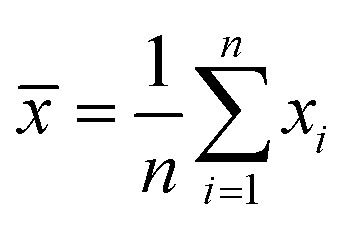

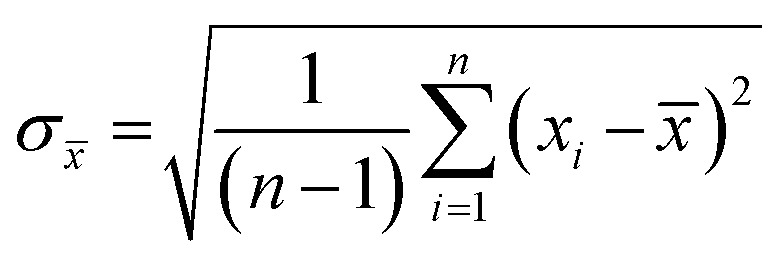


## Results and discussion

### Microbial adaptation to syngas fermentation

The wild-type *C. ljungdahlii* was initially cultured in ATCC Medium 1754 with 5.0 g L^−1^ fructose and preserved with 20% glycerol at −80 °C (*Cl*_wt_). ALE was performed over 20 transfers (Fig. 1B): transfers 1–9 involved gradual fructose reduction under constant syngas (25% CO, 10% H_2_, 65% CO_2_, 112 mL headspace), followed by 11 transfers using syngas exclusively. Each 72 hour transfer was conducted at 37 °C and 150 rpm, with OD_600_ monitored to confirm consistent growth (Fig. S3 and S4†). The final adapted culture was designated *Cl*_adapt_.

To validate the success of ALE, we assessed bacterial growth on syngas under batch and continuous flow conditions (Fig. S1†), simulating potential future application designs.^24^ Cell populations were monitored *via* OD_600_ readings, and C_2_ products (acetate and ethanol) were quantified using quantitative ^1^H NMR (qNMR) spectroscopy (Fig. 1C and D). The adapted strain, *Cl*_adapt_, exhibited faster growth (Fig. S5†) and higher final OD_600_ values than the wild-type *Cl*_wt_ in both modes. In batch mode, *Cl*_adapt_ reached an OD_600_ increase (ΔOD_600_) of 0.043 ± 0.002 by day 4, approximately 2.5 times higher than the 0.017 ± 0.003 ΔOD_600_ of *Cl*_wt_. In continuous flow mode, by day 6, *Cl*_adapt_ and *Cl*_wt_ recorded OD_600_ values of 1.02 ± 0.34 and 0.09 ± 0.12, respectively, indicating an 11-fold increase in the adapted strain. Growth rate analysis (Table S1†) showed that *Cl*_adapt_ had rates of 0.38 ± 0.02 per day in batch mode and 0.96 ± 0.11 per day in flow mode, compared to *Cl*_wt_'s slower rates of 0.26 ± 0.01 and 0.23 ± 0.22 per day, respectively (Fig. S6†). The enhanced growth of *Cl*_adapt_ under flow mode suggests improved syngas utilization following ALE, likely due to increased CO tolerance, as previous studies have shown high CO concentrations can impede growth rates.^25,26^

In batch mode (Fig. 1C), *Cl*_adapt_ produced 0.27 ± 0.01 mM acetate and 0.06 ± 0.01 mM ethanol over seven days, amounting to 0.05 ± 0.02 mM C_2_ compounds per day from syngas. In contrast, *Cl*_wt_ produced 0.06 ± 0.01 mM acetate and 0.05 ± 0.01 mM ethanol, equivalent to 0.02 ± 0.01 mM C_2_ compounds per day, approximately three-fold less than *Cl*_adapt_, consistent with the observed growth rate differences. Under continuous flow mode (Fig. 1D), *Cl*_adapt_ generated 69.1 ± 28.3 mM acetate and 7.7 ± 5.2 mM ethanol in six days, translating to 12.8 ± 5.6 mM C_2_ compounds per day. In contrast, *Cl*_wt_ generated 0.54 ± 0.70 mM acetate and 0.09 ± 0.03 mM ethanol, or 0.11 ± 0.12 mM C_2_ compounds per day, approximately 120-fold lower than *Cl*_adapt_. This large discrepancy between these two modes of culture suggests that continuous flow conditions, providing sustained syngas availability, greatly enhance the performance of *Cl*_adapt_. Notably, under batch mode, ethanol production between the two strains showed no significant differences. However, under flow mode, the *Cl*_adapt_ strain produced 85 times more ethanol than *Cl*_wt_, which may be attributed to the higher availability of reducing substrates (*i.e.*, CO and H_2_) in continuous flow conditions, facilitating the conversion of acetate into ethanol. Additionally, negative ethanol values observed in the first five days of continuous flow mode (Fig. 1D) resulted from the initial presence of ethanol in the growth medium and its subsequent consumption by the bacteria over time. These findings confirm the successful adaptation of *Cl*_adapt_ to syngas, demonstrating significantly higher C_2_ compound production, especially under continuous flow operation.

### Metabolic insights into carbon flux

**Fig. 2:**
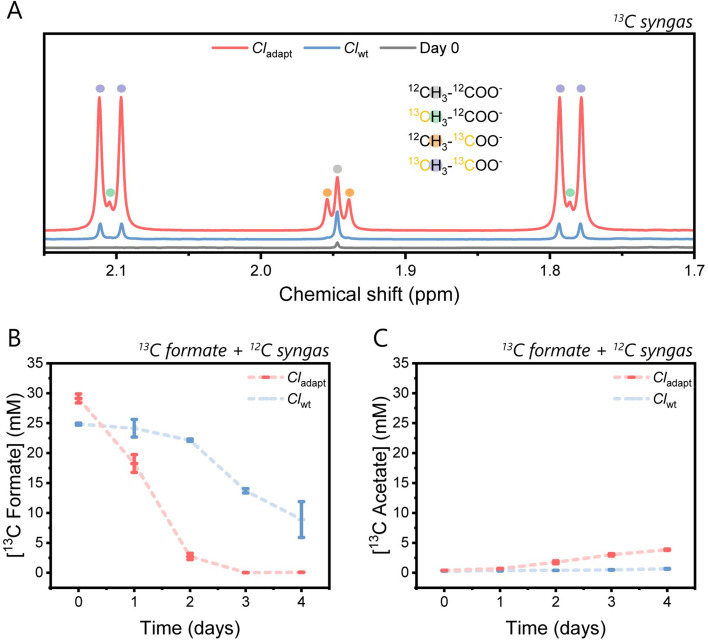
Growth analysis of strains using ^13^C- and ^13^C/^12^C mixed-substrates. (A) ^1^H qNMR spectra showing acetate production by *Cl*_adapt_ and *Cl*_wt_, when cultivated on ^13^C-syngas (112 mL headspace) over 12 days. The isotopologues are color-coded based on the position of the ^13^C atoms, as methyl or carboxyl, in acetate. (B) Consumption of ^13^C-formate, and (C) production of ^13^C-acetate, when cultivated on ^13^C-formate (∼30 mM) alongside ^12^C-syngas (112 mL headspace) over 4 days. Growth conditions: ATCC Medium 1754 (pH 5.9), 37 °C with 150 rpm mixing.

To confirm the source of carbon in the C_2_ products (Fig. 1A), we performed isotopic labelling experiments using ^13^C-syngas as the sole substrate for *Cl*_adapt_ and *Cl*_wt_ over 12 days. The isotopologues were analyzed by qNMR spectroscopy (Fig. 2A, S7 and Tables S2, S3† for acetate, and Fig. S8 and Table S4, S5† for ethanol). *Cl*_adapt_ and *Cl*_wt_ showed acetate (38.2 and 5.3 mM) containing mixtures of ^13^C and ^12^C, including ^13^CH_3_–^13^COO^−^, ^13^CH_3_–^12^COO^−^, ^12^CH_3_–^13^COO^−^, and ^12^CH_3_–^12^COO^−^. However, only *Cl*_adapt_ produced ethanol (0.7 mM) containing mixtures of ^13^CH_3_–^13^CH_2_OH and ^12^CH_3_–^13^CH_2_OH. For further discussion, see ESI Note 1.† The ^13^C/^12^C ratios were 86 : 14 for acetate and 95 : 5 for ethanol in *Cl*_adapt_, and 70 : 30 for acetate in *Cl*_wt_, indicating that most of the carbon in the products originates from ^13^C-syngas, with a smaller portion of ^12^C likely coming from the parental culture or cellular carbon reserves. This finding confirms that both *Cl*_adapt_ and *Cl*_wt_ can produce C_2_ compounds by converting the carbon in syngas. The higher ^13^C content and the presence of ethanol as a more reduced product in *Cl*_adapt_ indicates better syngas uptake and conversion activity than *Cl*_wt_.^27^

Notably, both strains produced less ^13^CH_3_–^12^COO^−^ than ^12^CH_3_–^13^COO^−^ (Table S3†), a metabolic preference that extended to ^12^CH_3_–^13^CH_2_OH (Table S5†). This suggests a preference for retaining the ^12^C-methyl rather than ^12^C-carboxyl group. Compared to *Cl*_wt_ (^13^CH_3_–^12^COO^−^/^12^CH_3_–^13^COO^−^ = 0.9), the *Cl*_adapt_ (^13^CH_3_–^12^COO^−^/^12^CH_3_–^13^COO^−^ = 0.5) shows a more unbalanced ^12^C/^13^C distribution. This observation may reflect a bottleneck in the *Cl*'s Wood–Ljungdahl pathway's methyl branch,^28^ which *Cl*_adapt_ has not yet overcome, or alternatively the carbonyl branch has been significantly enhanced during adaptation.

To investigate differences in the methyl branch of the Wood–Ljungdahl pathway between *Cl*_adapt_ and *Cl*_wt_, we performed a second isotopic labeling experiment using ^13^C-formate and ^12^C-syngas. *Cl*_adapt_ exhibited significantly faster growth, achieving a cell population five times greater than *Cl*_wt_ within four days (Fig. S9A†). *Cl*_adapt_ consumed ∼25 mM of ^13^C-formate within two days, whereas *Cl*_wt_ utilized only ∼15 mM over four days (Fig. 2B), resulting in a markedly higher production of ^13^C-acetate in *Cl*_adapt_ (3.86 ± 0.11 mM *vs.* 0.66 ± 0.06 mM) (Fig. 2C). This finding highlights *Cl*_adapt_'s enhanced efficiency in utilizing formate for such as growth and acetate production, reflecting optimized pathway dynamics compared to *Cl*_wt_. For further discussion, see ESI Note 2 (Fig. S9 and 10).†

### Whole genome sequencing

We conducted whole genome sequencing analysis to investigate the genetic changes in *Cl*_adapt_ compared to *Cl*_wt_. Whole genome sequencing of *Cl*_adapt_ and *Cl*_wt_ revealed several mutations compared to the reference genome in the NCBI database (Table S6, S7 and Fig. S11†). Notably, some mutations were present in both strains, suggesting that the wild-type strain had already diverged from the reference genome prior to our study. Additionally, mutations identified exclusively in *Cl*_adapt_ may have emerged either through the ALE process or as random variations in the single colony selected for sequencing. Similarly, the presence of unique mutations in *Cl*_wt_ suggests possible genetic drift or reversion events. Given these observations, future studies should validate key mutations through Sanger sequencing of multiple colonies to distinguish true evolutionary changes from colony-specific variations.

Notwithstanding these limitations—including the restricted sample numbers and incomplete functional annotation of some *C. ljungdahlii* genes—the molecular differences observed between *Cl*_adapt_ and *Cl*_wt_ suggest that the strains are distinct at a genetic level. While these differences may partly explain the variations observed in growth and C_2_ production, further studies are needed to establish a definitive link. For an in-depth analysis of the mutations differentiating *Cl*_adapt_ from *Cl*_wt_, please refer to ESI Note 3.

### Photocatalytic domino valorization

**Fig. 3:**
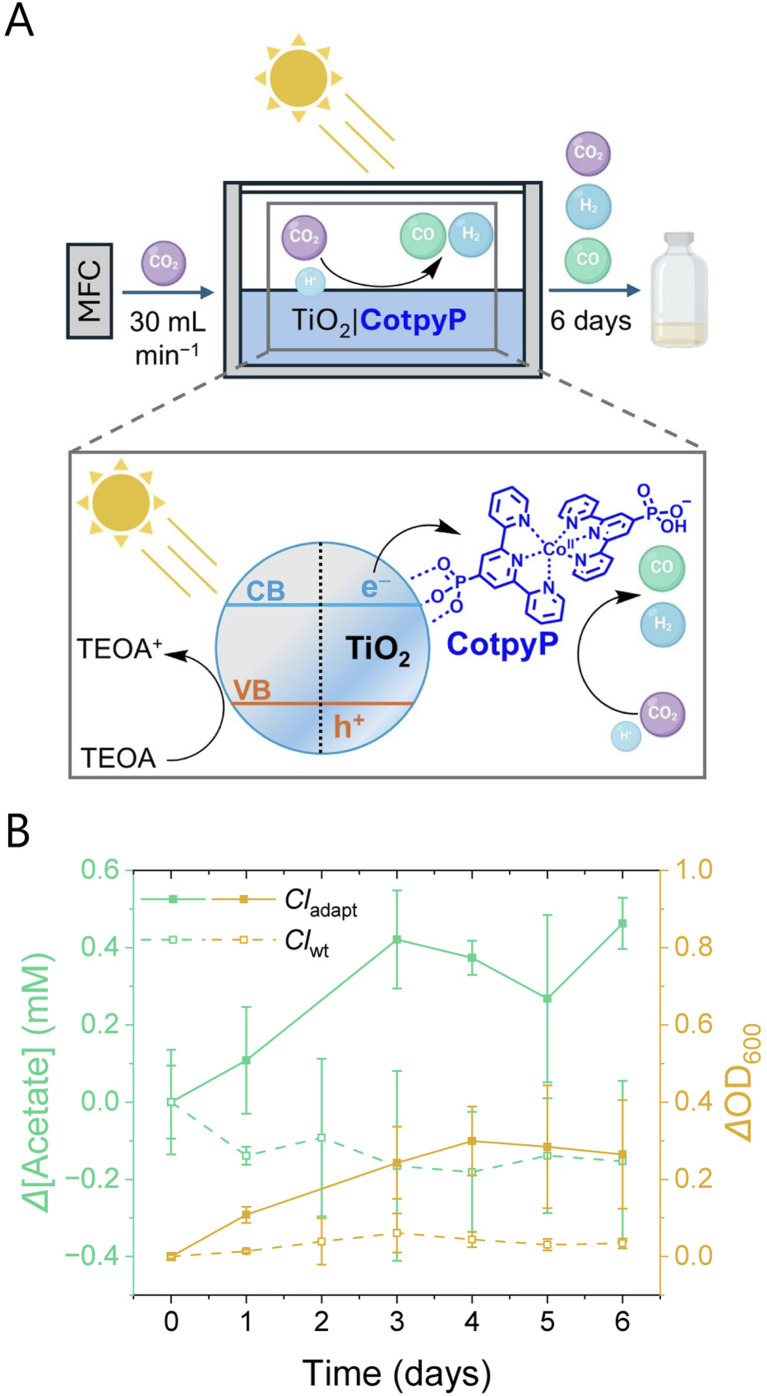
Light-driven CO_2_ domino valorization system. (A) Scheme of flow mode setup: the mass flow controller (MFC) purges CO_2_ through the photoreactor, where TiO_2_|CotpyP generates photocatalytically syngas in CO_2_-saturated water containing TEOA (0.1 M). The photoreactor is irradiated by filtered simulated sunlight from the top; and the syngas output flows then through the bioreactor where bacteria are cultured. (B) Production of acetate and biomass by *Cl*_adapt_ (solid line) and *Cl*_wt_ (dotted line) in the bioreactor when connected to the photocatalytically produced syngas over 6 days, expressed as variation of acetate concentration and optical density (OD_600_).

To source syngas for microbial fermentation directly from solar CO_2_ conversion, we scaled up a previously reported photocatalytic system composed of light-absorbing TiO_2_ nanoparticles (P25) and a phosphonated cobalt(ii)(terpyridine)_2_ CO_2_ reduction molecular catalyst (CotpyP, 20 μmol g_TiO_2__^−1^; Fig. 3).^21,29^ The choice of this photocatalytic semiconductor-molecule hybrid system was based on its suitability for scale-up to produce sufficient syngas quantities and coupling with aqueous microbial fermentation: it consists of Earth-abundant elements and readily available light absorber (TiO_2_) and co-catalyst (CotpyP) components in the required quantities. The photocatalyst system is also robust and can be easily repaired by addition of fresh CotpyP (see Experimental Section). It is therefore versatile and well suited for longer-term and multi-cycle experiments for aqueous CO_2_ photoreduction under batch^30^ and flow conditions to couple with biocatalysis (see below; Table S8†).

We readily scaled-up this hybrid TiO_2_|CotpyP photocatalyst from 5 mg to 365 mg without a significant effect in its catalytic activity during photocatalysis in the presence of triethanolamine (TEOA, 0.1 M) as sacrificial electron donor in water (0.22 L) using filtered simulated solar irradiation (see Experimental Section). The photoreactor used for scaling-up is cylindrical, with a surface area of 217 cm^2^ and a volume of 1.5 L (Fig. S2†). This photoreactor features a transparent top window that allows the simulated sunlight to photoexcite the semiconductor TiO_2_ nanoparticles, leading to electron transfer from TiO_2_ to the catalyst CotpyP. The reduced CotpyP reacts with aqueous CO_2_ resulting in the formation and release of CO and H_2_ gaseous products. Further details on the catalytic CO_2_ reduction mechanism of TiO_2_-bound CotpyP can be found in a previous report.^21^ The positive charges (holes) formed in TiO_2_ are neutralized through the oxidation of the sacrificial electron donor TEOA present in the solution, which regenerates the original ground state of the semiconductor and allows the photocatalytic cycle to repeat.

Finally, we coupled the scaled-up synthetic photocatalytic CO_2_ reduction system to the syngas-fermenting *Cl*_adapt_, creating an inorganic-bacterial system capable of domino valorization of CO_2_ into acetate and biomass with solar energy (Fig. 3A). The aqueous TiO_2_|CotpyP photocatalyst suspension (0.22 mL) was exposed to a flow of CO_2_ over six days (flow mode), mimicking the flow conditions described above for fermentation, and the photogenerated syngas was continuously flowed from the photoreactor to the bioreactor (Fig. 3A; see also ESI Note 4 for batch mode experiment details).

In flow mode, TiO_2_|CotpyP produced syngas under irradiation and exhibited activities of 148 ± 97 nmol CO g_TiO_2__^−1^ min^−1^ and 367 ± 606 nmol H_2_ g_TiO_2__^−1^ min^−1^ (Table S9†). The generated solar syngas had thus a CO : H_2_ ratio of ∼30 : 70 and TiO_2_|CotpyP under flow conditions produced 1.3 mmol of CO after 6 days of continuous experiment. Under dark conditions, TiO_2_|CotpyP was unable to produce syngas. Compared to other state-of-the-art photocatalytic flow systems, such as ZnSe–BF_4_|cobalt porphyrin, which produces 19 × 10^−3^ mmol of CO after 17 h,^30^ and Bi_2_WO_6_, which produces 1.2 × 10^−2^ mmol of CO after 4 h,^31^ TiO_2_|CotpyP exhibits a very high yield (Table S8†).

Syngas produced by TiO_2_|CotpyP allowed the *Cl*_adapt_ cultures to increase their biomass from the start of the flow experiment until day 4, reaching a ΔOD_600_ of 0.30 ± 0.09, which remained approximately constant until day 6 (Fig. 3B). In contrast to the batch mode (Fig. S12†), the flow configuration showed a rise in acetate from day 0 to day 6, with a maximum Δ[acetate] of 0.46 ± 0.07 mM (Fig. 3B). Under identical experimental conditions, *Cl*_wt_ exhibited significantly lower performance, with a ΔOD_600_ of only 0.03 ± 0.01 and no acetate production after 6 days. The lack of ethanol production, lower ΔOD_600_, and Δ[acetate] for *Cl*_adapt_ can be attributed to the low syngas concentration (<0.1%) in the photocatalysis gas stream, as well as the slower formation rate by TiO_2_|CotpyP compared to direct feeding with a premixed syngas cylinder. As shown in Fig. 1D, *Cl*_adapt_ achieves high biomass and C_2_ product outputs under 10 mL of syngas per minute (which equals to 102 μmol CO min^−1^ and 41 μmol H_2_ min^−1^), highlighting the limitations of the presented photocatalytic configuration and the need for improved systems capable of delivering higher syngas formation rates to support *Cl*_adapt_.

Our results demonstrate that simple photocatalytic powder systems can be readily scaled up and, in principle, be used to supply solar syngas directly to gas-fermenting adapted bacteria. This approach offers an alternative to wired devices such as electrolyzers and photoelectrochemical cells.^3,5,6,11^ Furthermore, these findings highlight the need for innovative renewable energy-driven syngas production systems and device architectures—such as advanced electrolyzers and photoelectrochemical systems—for researchers in biohybrids and domino catalysis. Future systems should be designed to bridge the potential mismatch between syngas production and fermentation rates, maximizing the capabilities of gas-fermenting adapted bacteria to sustain high levels of C_2_ production and cell growth over extended periods.

## Conclusions

The reported proof-of-concept semi-biological system demonstrates the integration of photocatalytic CO_2_ reduction with adapted bacterial biosynthesis. Unlike previous nanomaterial–bacteria biohybrid systems that relied on ill-defined charge transfer pathways or photogenerated H_2_, our approach employs direct ALE-derived adaptation to achieve high C_2_ product yields solely from syngas produced from solar CO_2_ reduction. This work illustrates the scalability of photocatalytic powder systems and underscores the simplicity of implementing adaptive evolution in a chemistry laboratory, thereby enhancing the microorganisms' natural capabilities while seamlessly coupling them with inorganic catalytic systems. While our sequencing analysis of the ALE-derived strain has limitations—stemming from restricted sample numbers and incomplete functional annotation—it provides initial insights into the genomic changes that may underpin the observed phenotypic improvements. Future investigations will be essential to pinpoint the key genes responsible for faster growth and higher product yields, as well as to optimize these biological–inorganic hybrid systems by improving syngas formation rates, eliminating the sacrificial electron donor in the photocatalysis process, and advancing genetic and metabolic engineering strategies. Overall, this study highlights the transformative potential of combining innovations from biology, materials science, and chemistry to address critical global environmental challenges.

## Author contributions

Lin Su conceptualization, data curation, formal analysis, funding acquisition, investigation, visualization, methodology, project administration, writing – original draft, review & editing; Santiago Rodríguez-Jiménez conceptualization, data curation, formal analysis, funding acquisition, investigation, visualization, methodology, project administration, writing – original draft, review & editing; Marion I. M. Short investigation, methodology, writing – review & editing; Erwin Reisner conceptualization, resources, formal analysis, supervision, funding acquisition, validation, investigation, visualization, project administration, writing – original draft, review & editing.

## Conflicts of interest

There are no conflicts to declare.

## Supplementary Material

SC-016-D5SC00764J-s001

## Data Availability

Experimental data supporting the findings of this study are available from the University of Cambridge data repository (https://doi.org/10.17863/CAM.118249).
