# Supplementary material for: Adapting gas fermenting bacteria for light-driven domino valorization of CO2
Source: Chem Sci. 2025 May 12;16(26):11801–8. doi: 10.1039/d5sc00764j (PMC12123536; doi:10.1039/d5sc00764j)
Supplement: SC-016-D5SC00764J-s001 [file SC-016-D5SC00764J-s001.pdf]

Supplementary Information (SI) for Chemical Science.
This journal is © The Royal Society of Chemistry 2025

Supporting Information for

# Adapting Gas Fermenting Bacteria for Light-driven Domino Valorization of $CO_2$

Lin Su[1,2,‡], Santiago Rodríguez-Jiménez[1,‡], Marion I. M. Short[1], Erwin Reisner[1,*]

[1]Yusuf Hamied Department of Chemistry, University of Cambridge, Cambridge, U.K.

[2] Present address: School of Biological and Behavioural Sciences, Queen Mary University of London, Mile End Road, London E1 4NS, U.K.

[‡]These authors contributed equally to this work.

*E-mail: reisner@ch.cam.ac.uk

## *Supplementary Notes*

**Supplementary Note 1 regarding isotopic labelling experiments using $^{13}C$-syngas.** The $^{13}C$-coupled proton constants for acetate are $^{1}J_{CH}$ = 127.4 and $^{2}J_{CH}$ = 6.0 Hz (**Figure 2A**)[1], and for ethanol are $^{1}J_{CH}$ = 143.0 and $^{2}J_{CH}$ = 2.5 Hz (**Figure S6**)[2]. Analysis of the qNMR spectra (**Figure S7**) for *Cl*$_{adapt}$ showed distributions for acetate of 80% $^{13}CH_3$-$^{13}COO^-$, 4% $^{13}CH_3$-$^{12}COO^-$, 9% $^{12}CH_3$-$^{13}COO^-$, and 7% $^{12}CH_3$-$^{12}COO^-$ (**Tables S2 and S3**). For ethanol, the distributions were 90% $^{13}CH_3$-$^{13}CH_2OH$ and 10% $^{12}CH_3$-$^{13}CH_2OH$ (**Figure S7**, **Tables S4 and S5**). In contrast, *Cl*$_{wt}$ exhibited 68% $^{13}CH_3$-$^{13}COO^-$, 1.6% $^{13}CH_3$-$^{12}COO^-$, 1.8% $^{12}CH_3$-$^{13}COO^-$, and 29% $^{12}CH_3$-$^{12}COO^-$, with no ethanol.

**Supplementary Note 2 regarding isotopic labelling experiments using $^{13}C$-formate and $^{12}C$-syngas.** $OD_{600}$ measurements revealed that *Cl*$_{adapt}$ exhibited significantly faster growth than *Cl*$_{wt}$, achieving a cell population five times greater within four days (**Figure 2B**), consistent with previous observations of accelerated growth on syngas. *Cl*$_{adapt}$ consumed ~25 mM of $^{13}C$-formate within two days, while *Cl*$_{wt}$ utilized only ~15 mM over four days, showing a slower initial rate likely due to downstream bottlenecks (e.g., formate to acetyl-CoA conversion) (**Figure 2C**). After four days, *Cl*$_{adapt}$ produced 3.86 ± 0.11 mM of $^{13}C$-acetate, compared to 0.66 ± 0.06 mM by *Cl*$_{wt}$, indicating a 6-fold increase in acetate production by the adapted strain (**Figure 2D**). This result underscores *Cl*$_{adapt}$'s enhanced efficiency in utilizing formate for such as growth and acetate production, likely reflecting optimized pathway dynamics.

Analysis of $^{12}C$-syngas conversion showed both $^{12}C$-formate and $^{12}C$-acetate production during growth (**Figure S9B, C**). Although *Cl*$_{wt}$ initially produced $^{12}C$-formate at a slower rate (1.5 mM $day^{-1}$ during the first 2 days), its production rates eventually increased to 6 mM $day^{-1}$ between day 2 and day 4, matching the rate observed in *Cl*$_{adapt}$ (6 mM $day^{-1}$ in first 2 days). This suggests that formate synthesis from syngas is not the limiting step in the *Cl*$_{wt}$. After four days, *Cl*$_{adapt}$ produced 23.63 ± 0.11 mM of $^{12}C$-acetate, compared to 3.34 ± 0.14 mM by *Cl*$_{wt}$, indicating a 7-fold increase (**Figure 2D**). The *Cl*$_{adapt}$ strain also showed a 6-fold increase in utilizing $^{13}C$-formate, indicating a more efficient overall turnover in the pathway. qNMR analysis revealed that *Cl*$_{wt}$ predominantly synthesized $^{13}C$-acetate as $^{13}CH_3$-$^{12}COO^-$ (**Figure S10**), while *Cl*$_{adapt}$ produced both $^{13}CH_3$-$^{12}COO^-$ and $^{12}CH_3$-$^{13}COO^-$, suggesting that $^{13}C$-formate might be converted back to $^{13}CO_2$ or $^{13}CO$, contributing to the carbonyl branch. These findings are consistent with the earlier observations of $^{12}C/^{13}C$ distribution when using $^{13}C$-syngas. The rapid decrease in $^{12}C$-formate in *Cl*$_{adapt}$ between days 2 and 3, matching the depletion of $^{13}C$-formate by day 2, further indicates efficient substrate conversion in the adapted strain. Overall, these results demonstrate that *Cl*$_{adapt}$ has a more robust and efficient Wood-Ljungdahl pathway compared to *Cl*$_{wt}$, particularly in the conversion of formate, supporting its enhanced growth and $C_2$ product generation on syngas.

**Supplementary Note 3 regarding sequencing.**

*Genomic mutations in Cl$_{adapt}$ and Cl$_{wt}$*

We conducted whole genome sequencing (WGS) to compare *Cl*$_{adapt}$ with *Cl*$_{wt}$, aiming to link the observed phenotypic variations to genomic alterations relative to the reference sequence (NC_014328.1) listed in National Center for Biotechnology Information (NCBI) database. The analysis revealed a total of 41 mutations, with 8 unique to *Cl*$_{adapt}$, associated with 6 genes and 2

non-coding regions (**Table S6, S7, Figure S11**); conversely, *Cl*$_{wt}$ exhibited 17 unique mutations impacting 2 genes, alongside 16 mutations common to both strains, affecting 7 genes (**Table S6**).

Among the six gene mutations (**Table S7**) identified in *Cl*$_{adapt}$, two are classified as silent mutations, which are not expected to alter the amino acid sequence or protein structure but may still influence protein synthesis rate, folding, and function due to codon usage differences. While these two genes are not part of the core metabolic pathways such as the Wood–Ljungdahl pathway, they may still have indirect effects on bacterial growth and metabolism. Specifically, *CLJU_RS04595*, a sugar-specific transcriptional regulator, could influence carbohydrate uptake and regulatory networks that impact growth under certain conditions. Likewise, *CLJU_RS09515*, which is linked to phenazine biosynthesis, might modulate redox balance or stress responses, thereby affecting metabolic efficiency. However, further functional studies are needed to clarify the precise roles of these genes in *Clostridium ljungdahlii*'s growth and central metabolism during syngas fermentation. Of the remaining mutations, one affects gene *ClJU_RS00445*, which encodes the deoxyribose-phosphate aldolase. This enzyme catalyzes the conversion of deoxyribose-phosphate aldolase into glyceraldehyde 3-phosphate and glyoxylate, and serves as a crucial link between nucleic acid and carbohydrate metabolism[3,4]. Another mutation occurred in the *grpE* gene, which acts as a nucleotide exchange factor essential for the regulation of the protein folding machinery and the heat shock response[5]. Additionally, a mutation was found in *ClJU_RS06845*, associated with an oligopeptide transporter. This transporter is not only pivotal for nutrient uptake but also plays a role in the internalization of signaling peptides involved in quorum-sensing pathways[6]. Lastly, a mutation in *ClJU_RS11365* affects a molybdenum-iron protein of nitrogenase, responsible for catalyzing the process of nitrogen fixation, a key biochemical reaction for nitrogen assimilation.[7]

In *Cl*$_{wt}$, we identified 17 unique mutations affecting two genes (**Table S7**). One of these genes, *CLJU_RS07315*, encodes the 16S ribosomal RNA. While the 16S rRNA gene is typically conserved, mutations can occur due to environmental pressures. BLAST analysis (**Figure S14**) indicates that *Cl*$_{wt}$ aligns closely with *Clostridium ljungdahlii* and its relative *Clostridium autoethanogenum*, suggesting that contamination is unlikely. The second gene *CLJU_RS14955*, which has been found to have multiple changes with both *Cl*$_{wt}$ and *Cl*$_{adapt}$ strains (**Table S6**), encodes a protein containing a Lamin Tail Domain (LTD). LTDs are conserved globular regions found in lamins, which are structural proteins of the nuclear lamina involved in maintaining nuclear integrity and regulating various nuclear functions. However, the specific function of this LTD-containing protein in *Clostridium ljungdahlii* remains unclear.

While clear evidence linking these mutations directly to the enhanced growth rate of the adapted strain *Cl*$_{adapt}$ under syngas fermentation and its increased production of $C_2$ compounds is lacking, the identified genomic alterations support the adaptation process of *Clostridium ljungdahlii* to syngas across 20 transfers. These changes contribute to understanding the genetic underpinnings of the observed phenotypic differences.

**Supplementary Note 4 regarding photocatalytic batch mode with the inorganic-bacterial system and exclusion control experiment without solar light irradiation.** In batch mode, irradiated $TiO_2$|**CotpyP** produced 0.9 ± 0.4 mmol CO $g_{TiO2}^{-1}$ and 3.3 ± 1.1 mmol $H_2$ $g_{TiO2}^{-1}$ after 24 h (**Table 10**). This activity corresponds to a **CotpyP**-based turnover number (TON) for CO of 43 ± 18. After connecting the photoreactor with the bioreactor for 6 days, the bacteria consumed CO and $H_2$ down to 0.6 ± 0.3 mmol CO $g_{TiO2}^{-1}$ and 0.9 ± 1.4 mmol $H_2$ $g_{TiO2}^{-1}$ (**Table S9**), respectively.

This resulted in an increase of $OD_{600}$, from 0.10 ± 0.01 to 0.61 ± 0.29, and a decrease of acetate concentration from 1.45 ± 1.05 mM to 0.43 ± 0.55 mM (**Figure S12**). The concomitant consumption of syngas and the increase in biomass are in alignment with our results (**Figure 1**). The decrease in acetate over 6 days indicates that under our experimental conditions the bacterial cultures prioritized biomass production over $C_2$ products. In all cases, $CO_2$ containing 2% $CH_4$ was used as the internal gas chromatography standard, and the generated gaseous products, $H_2$ and CO, were monitored daily by analyzing the headspace using gas chromatography. The production of acetate and $OD_{600}$ were monitored daily using qNMR and UV-Vis spectroscopy, respectively.

Under dark conditions, $TiO_2$|**CotpyP** was unable to produce syngas. Nonetheless, bacteria were still able to increase their biomass over 6 days, reaching a $\Delta OD_{600}$ of 0.36 ± 0.03 by depleting all residual acetate present in solution ($\Delta$[acetate] of –0.27 ± 0.04 mM) from the parental culture and cellular carbon reserves within the cells (**Figure S13**).

### ***Supporting Information Figures***

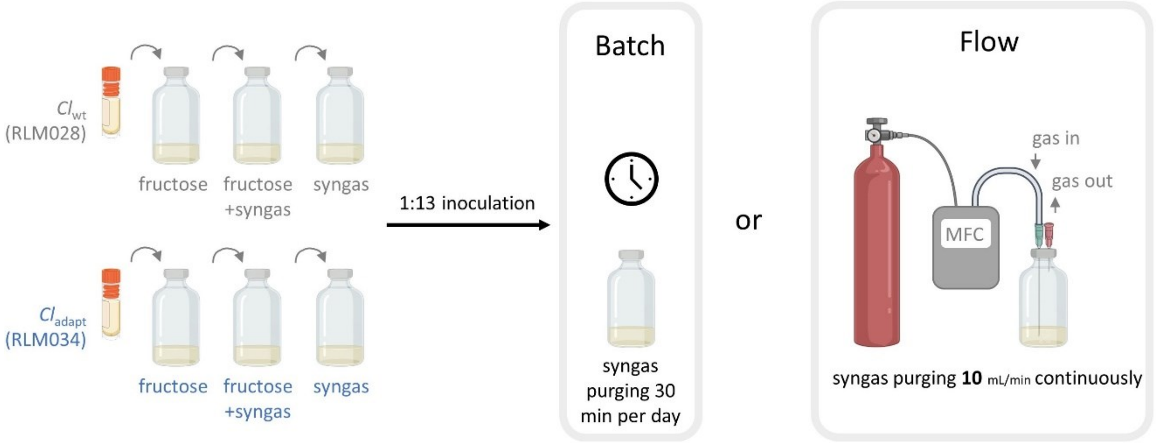


**Figure S1.** Depicts the schematic of the process of recovering strains from -80 °C stocks and testing under syngas growth under batch and continuous flow. For a fair comparison as the wildtype strain would not be able to grow with syngas directly, both $Cl_{wt}$ and $Cl_{adapt}$ strains were first recovered from –80 °C stocks in ATCC Medium 1754 containing fructose, supplemented with a 20% $CO_2$ and 80% $N_2$ gas mixture. Subsequently, the strains were transferred to ATCC Medium 1754 (13 mL) with 5.00 g $L^{-1}$ fructose and syngas (25% CO / 10% $H_2$ / 65% $CO_2$) in the headspace (112 mL), followed by a final transfer to ATCC Medium 1754 without fructose, exclusively utilizing syngas in the headspace. After these three-transfer recovery growth, cultures were subjected to growth experiments.

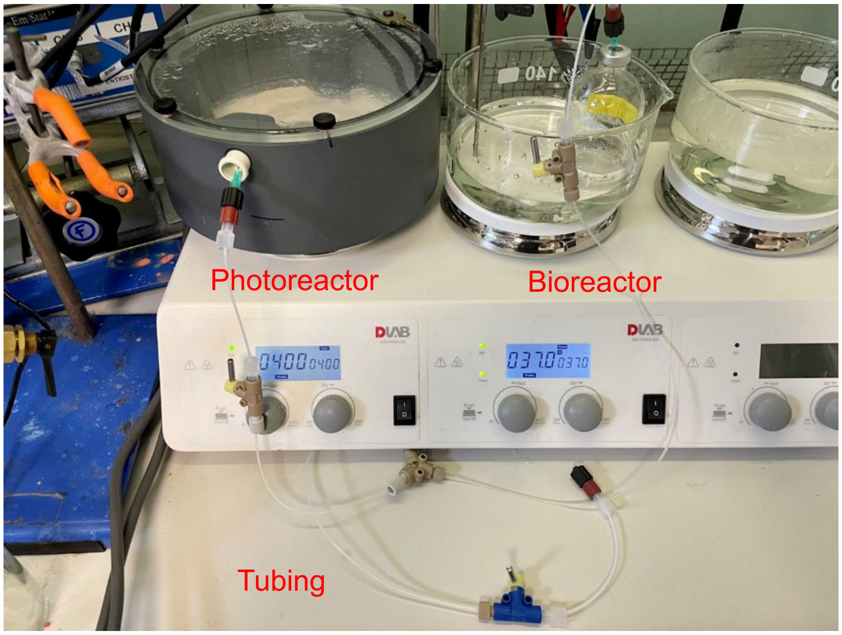


**Figure S2.** Picture of the scaled-up photoreactor setup during batch mode, where the photoreactor, irradiated for 24 h, is connected to the bioreactor for 6 days.

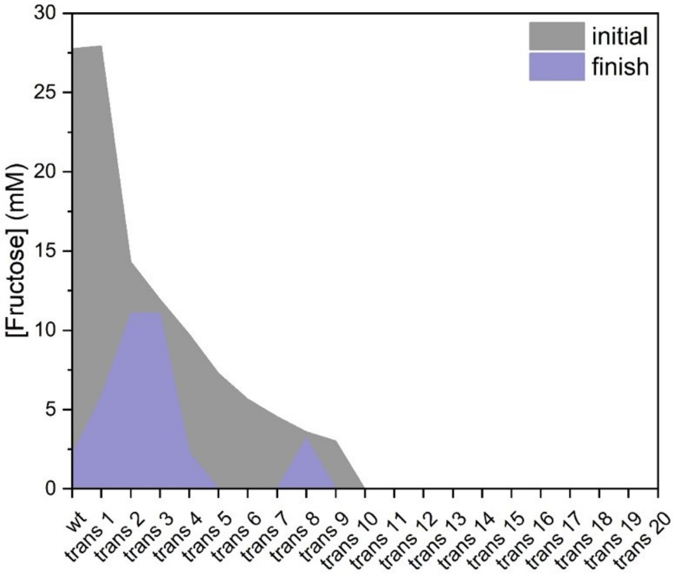


**Figure S3.** Fructose concentrations in the ATCC Medium 1754 during *C. ljungdahlii* adaptation growth. The initial concentration was estimated based on the dilution ratio from fructose stocks and residual fructose in the inoculum from previous growth, representing the level at inoculation onset; the final concentration was quantified using NMR at the end of the incubation period.

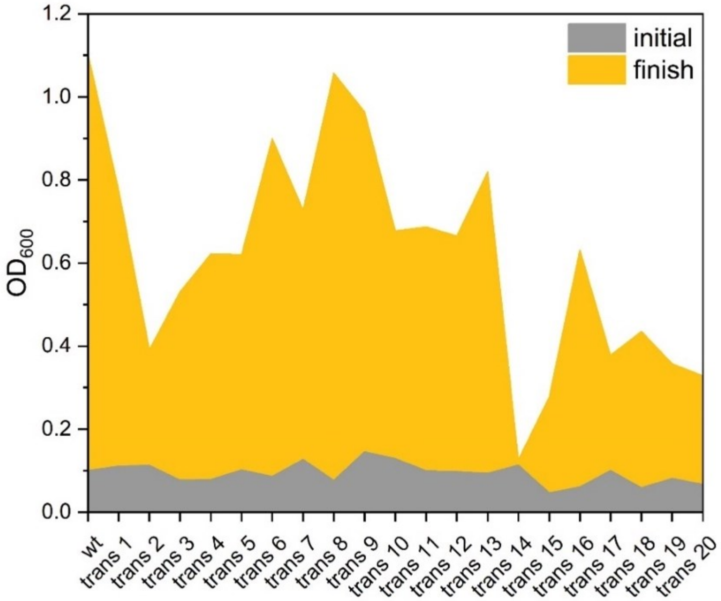


**Figure S4.** $OD_{600}$ variations during syngas adaptation across successive transfer growths.

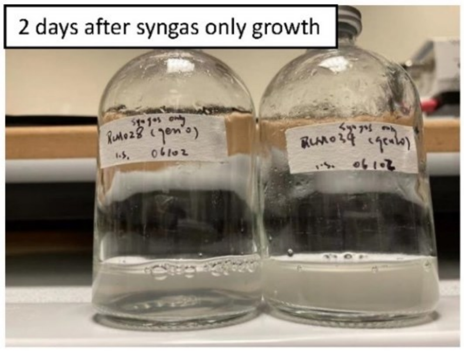


**Figure S5.** Photo of the bacterial growth in batch mode after 2 days (left: $Cl_{wt}$; right: $Cl_{adapt}$).

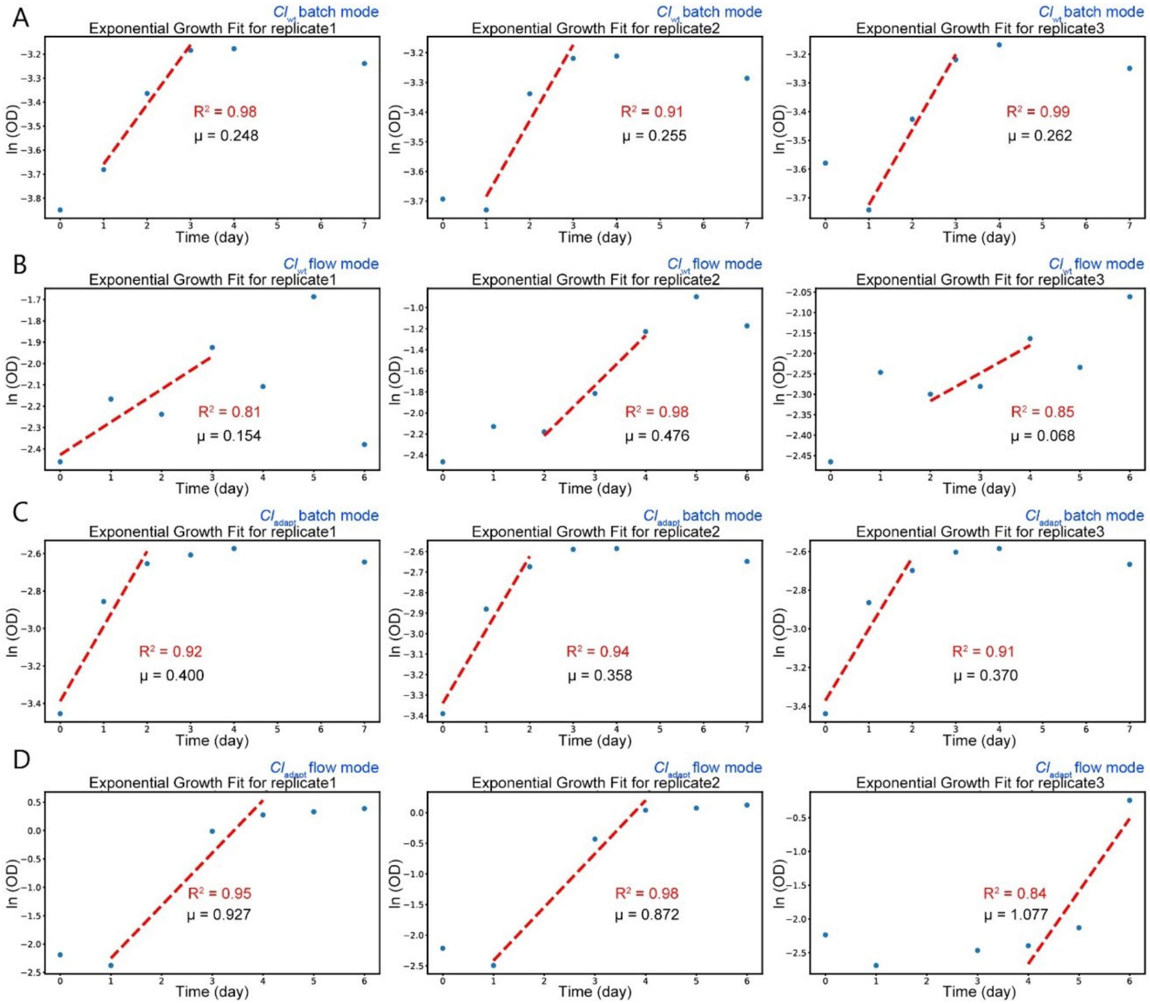


**Figure S6.** The growth rate calculations with flitting results. $Cl_{wt}$ growth under (A) batch syngas mode and (B) flow syngas mode. $Cl_{adapt}$ growth under (C) batch syngas mode and (D) flow syngas mode. The growth rate (μ) and coefficient of determination ($R^2$) are noted in each panel.

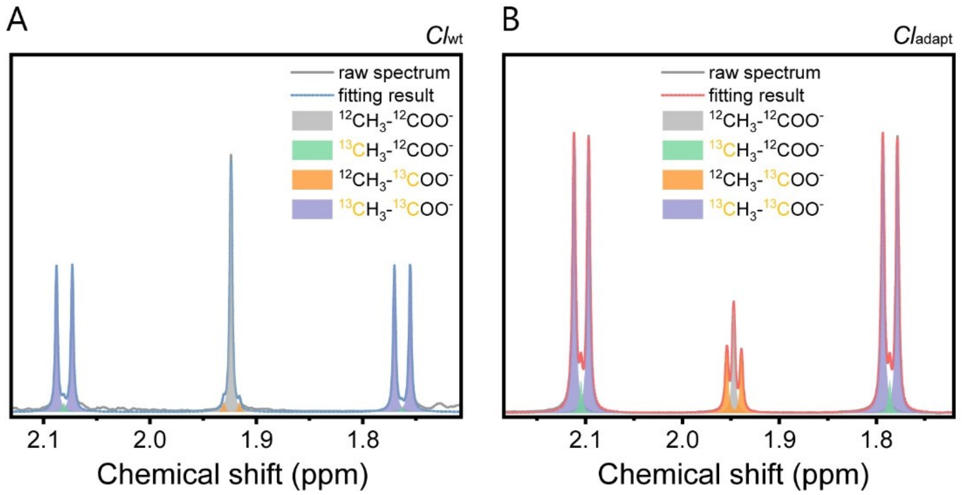


**Figure S7.** $^{1}H$ NMR spectra of acetate produced by wildtype strain *Cl*wt (A) and adapted strain *Cl*adapt (B). These spectra resulted from fermentation of a $^{13}CO_2/^{13}CO/H_2$ (65:25:10) gas mixture. Peak fitting was conducted using the Multiplet Analysis and Line Fitting functions in Mnova (version 15.0, Mestrelab).

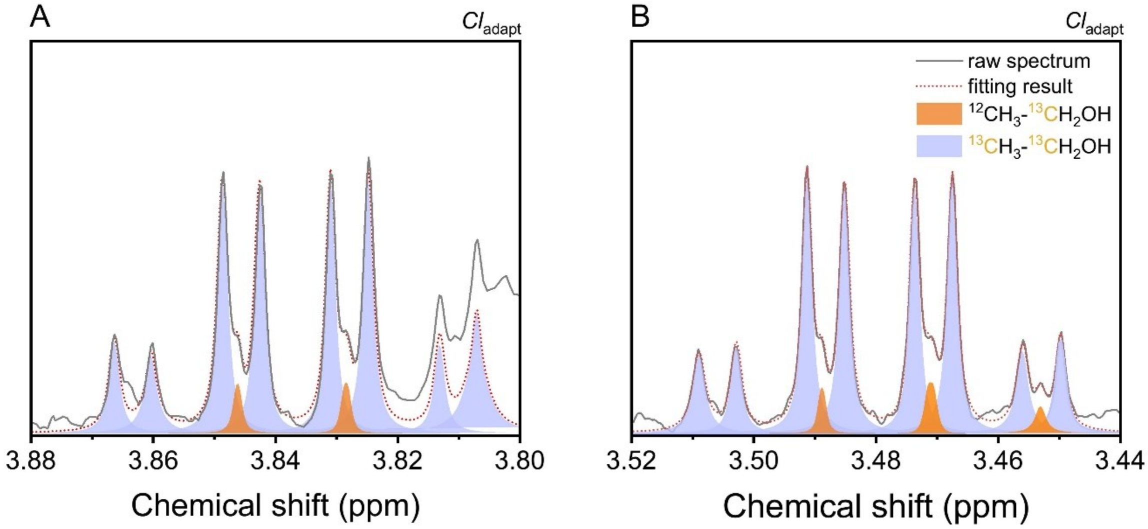


**Figure S8.** $^{1}H$ NMR spectra of ethanol's $CH_2$ peaks produced by the adapted strain *Cl*adapt. Panel (A) displays peaks between 3.86 and 3.81 ppm, and panel (B) shows peaks between 3.51 and 3.45 ppm. Ethanol's $CH_3$ peaks are not shown, as they could not be reliably distinguished and accurately peak-fitted. These spectra were obtained from fermentation using a $^{13}CO_2/^{13}CO/H_2$ (65:25:10) gas mixture. Peak fitting was conducted using the Multiplet Analysis and Line Fitting functions in Mnova (version 15.0, Mestrelab).

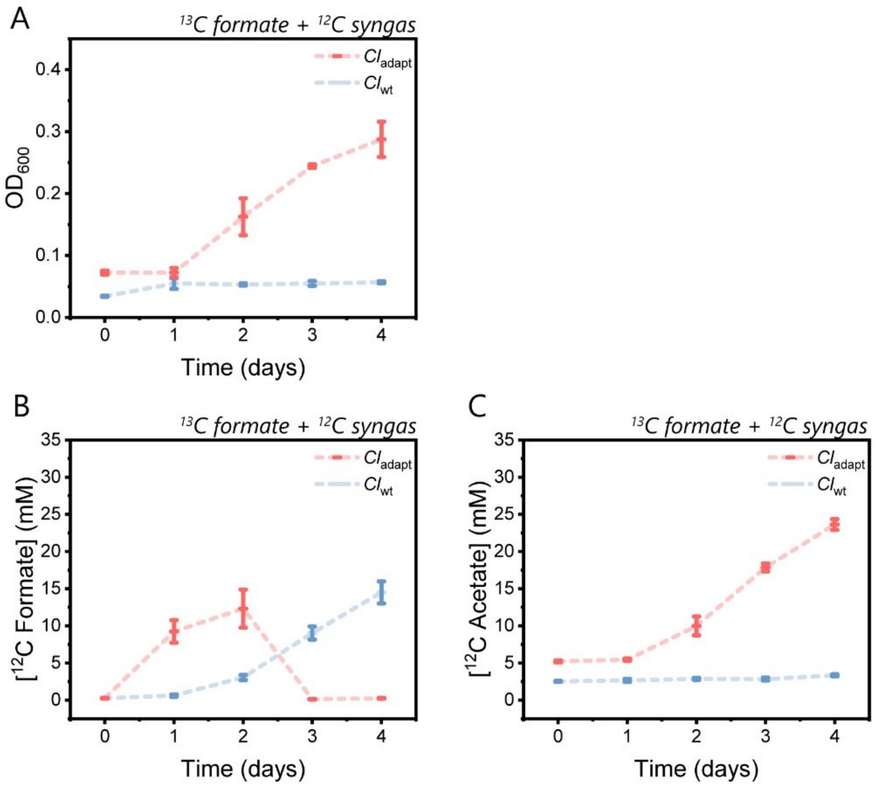


**Figure S9.** Bacteria growth (A), and resultant production of (B) $^{12}$C formate, (C) $^{12}$C acetate, when cultivated strains on $^{13}$C formate alongside $^{12}$C syngas.

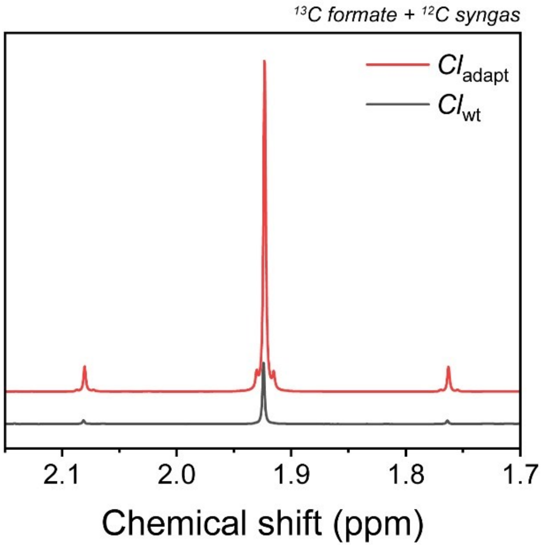


**Figure S10.** $^{1}$H NMR spectrum of acetate produced by wildtype strain $Cl_{wt}$ and adapted strain $Cl_{adapt}$. This spectrum results from fermentation of $^{13}$C formate with a $^{12}CO_2/^{12}CO/H_2$ (65:25:10) gas mixture.

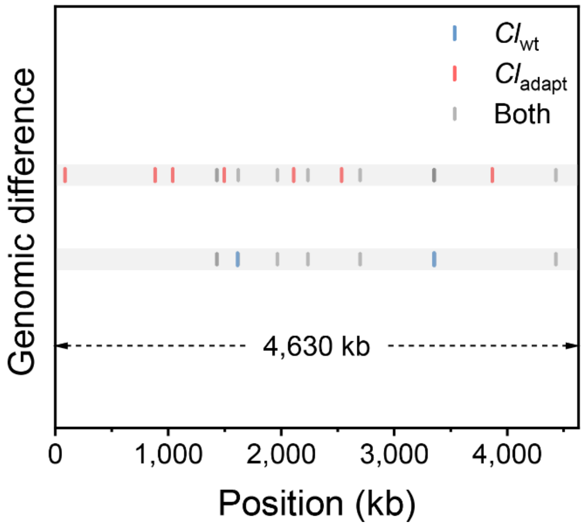


**Figure S11.** Visual Comparison of Genomic Variations in *Clostridium ljungdahlii* Strains Relative to the NCBI Reference Sequence. Mutations unique to the wildtype strain $Cl_{wt}$ are marked in blue, those unique to the adapted strain $Cl_{adapt}$ in red, and mutations shared by both strains in grey.

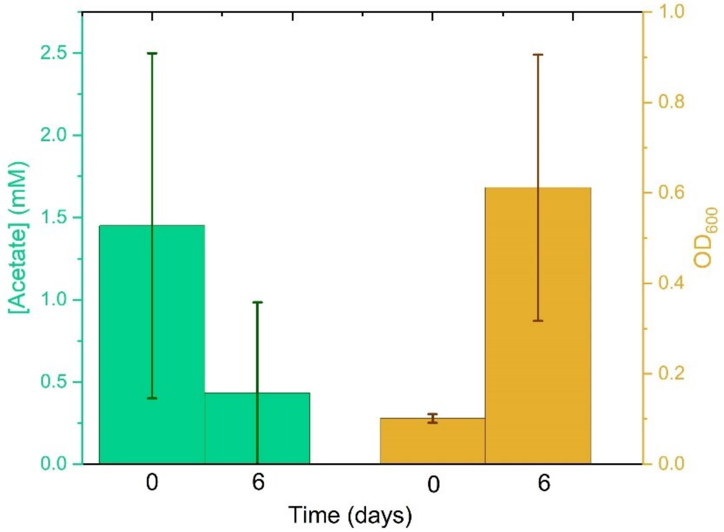


**Figure S12.** Performance of the Adapted Strain Coupled with Light-Driven Syngas Generation Systems under batch conditions. This figure shows the acetate concentration and optical density ($OD_{600}$) obtained when connecting in batch mode the bacteria cultures with the photoreactor containing $TiO_2$|**CotpyP** and 0.1 M TEOA over 6 days. $CO_2$ containing 2% $CH_4$ as internal standard was used to purge the photoreactor before starting solar light irradiation.

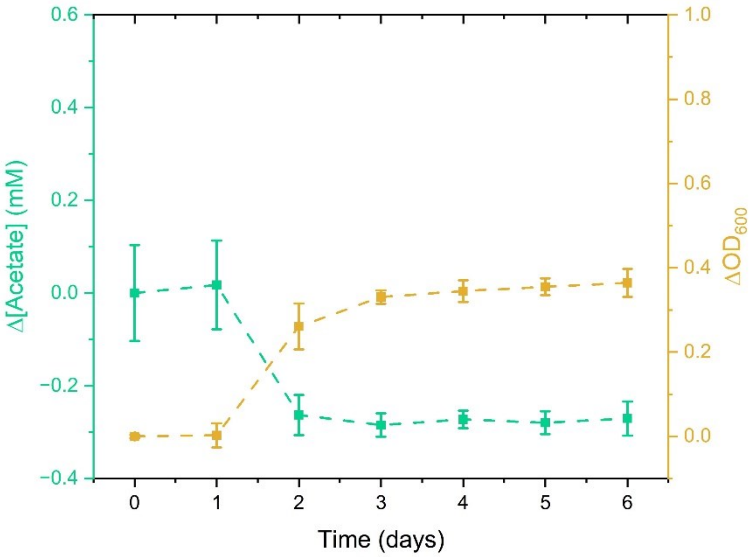


**Figure S13.** Performance of the Adapted Strain Coupled with the Light-Driven Syngas Generation System under Flow Conditions but No Solar Light Irradiation. This figure shows the acetate concentration and optical density ($OD_{600}$) obtained when connecting under flow the *Cl*$_{adapt}$ strain cultures with the photoreactor containing $TiO_2$|**CotpyP** and 0.1 M TEOA over 6 days and with no solar light irradiation. $CO_2$ containing 2% $CH_4$ as internal standard was used to purge the whole setup throughout the experiments.

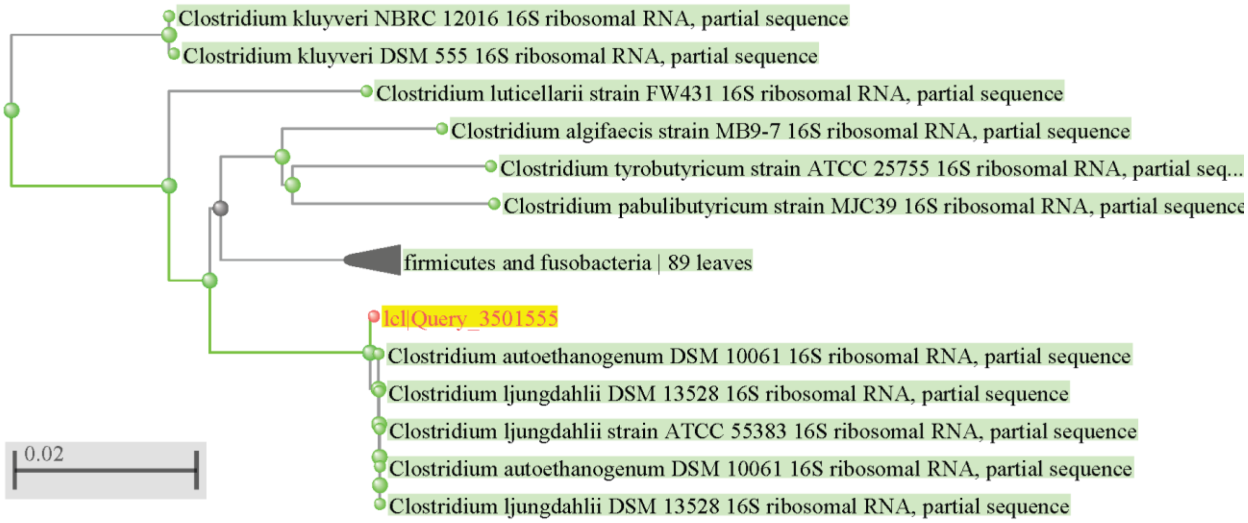


**Figure S14.** Distance tree of BLAST results for the mutated 16S rRNA sequence from *Cl*$_{wt}$, compared with closely related strains. This tree was generated using BLAST pairwise alignments. Query_3501555 is the mutated 16S rRNA sequence we submitted for BLAST.

## *Supporting Information Tables*

**Table S1.** Growth rate calculated during syngas growth. The $R^2$ reflects the calculated fitting error when processing the growth rate analysis. All errors represent the mean ± SD (n = 3 biological independent samples).

| Strain | Syngas mode | Growth rate ($day^{-1}$) | $R^2$ |
|---|---|---|---|
| ***Cl*wt** | batch | 0.26 ± 0.01 | 0.96 ± 0.04 |
| | flow | 0.23 ± 0.22 | 0.88 ± 0.09 |
| ***Cl*adapt** | batch | 0.38 ± 0.02 | 0.93 ± 0.02 |
| | flow | 0.96 ± 0.11 | 0.92 ± 0.07 |

**Table S2.** Detailed Summary of Acetate Peaks from $^1H$ NMR Spectra (Figure S7). This table lists the isotopic composition, chemical shift, and integrated areas of each peak. Analysis was performed using the Multiplet Analysis in Mnova (version 15.0, Mestrelab).

| Strain | Specific isotope molecule | Peak's chemical shift (ppm) | Integrated Area (%) |
|---|---|---|---|
| | $^{13}CH_3$-$^{13}COO^-$ | 2.09 | 17.0 |
| | $^{13}CH_3$-$^{12}COO^-$ | 2.08 | 1.1 |
| | $^{13}CH_3$-$^{13}COO^-$ | 2.07 | 16.8 |
| | $^{12}CH_3$-$^{13}COO^-$ | 1.93 | 1.0 |
| ***Cl*wt** | $^{12}CH_3$-$^{12}COO^-$ | 1.92 | 28.7 |
| | $^{12}CH_3$-$^{13}COO^-$ | 1.92 | 0.8 |
| | $^{13}CH_3$-$^{13}COO^-$ | 1.77 | 17.2 |
| | $^{13}CH_3$-$^{12}COO^-$ | 1.76 | 0.5 |
| | $^{13}CH_3$-$^{13}COO^-$ | 1.76 | 16.9 |
| | $^{13}CH_3$-$^{13}COO^-$ | 2.11 | 20.4 |
| | $^{13}CH_3$-$^{12}COO^-$ | 2.11 | 2.2 |
| | $^{13}CH_3$-$^{13}COO^-$ | 2.10 | 19.6 |
| | $^{12}CH_3$-$^{13}COO^-$ | 1.95 | 4.4 |
| ***Cl*adapt** | $^{12}CH_3$-$^{12}COO^-$ | 1.95 | 7.4 |
| | $^{12}CH_3$-$^{13}COO^-$ | 1.94 | 4.2 |
| | $^{13}CH_3$-$^{13}COO^-$ | 1.79 | 20.2 |
| | $^{13}CH_3$-$^{12}COO^-$ | 1.79 | 2.2 |
| | $^{13}CH_3$-$^{13}COO^-$ | 1.78 | 19.5 |

**Table S3.** Summary of Area Measurements for each acetate isotope and the $^{13}C/^{12}C$ Ratio. This table presents a detailed analysis of the area contributions from different isotopes, along with the calculated $^{13}C/^{12}C$ ratios. For acetate species $^{13}CH_3$-$^{12}COO^-$ and $^{12}CH_3$-$^{13}COO^-$, which contain mixed $^{12}C$ and $^{13}C$ isotopes, the quantities were divided equally when calculating the $^{13}C/^{12}C$ ratio.

| Strain | Specific isotope molecule | Area (%) per isotope | Specific carbon isotopes | $^{13}C/^{12}C$ Ratio |
|---|---|---|---|---|
| ***Cl*$_{wt}$** | $^{13}CH_3$-$^{13}COO^-$ | 68 | $^{13}C$ | 70:30 |
| | $^{13}CH_3$-$^{12}COO^-$ | 1.6 | $^{13/12}C$ | |
| | $^{12}CH_3$-$^{13}COO^-$ | 1.8 | | |
| | $^{12}CH_3$-$^{12}COO^-$ | 29 | $^{12}C$ | |
| ***Cl*$_{adapt}$** | $^{13}CH_3$-$^{13}COO^-$ | 80 | $^{13}C$ | 86:14 |
| | $^{13}CH_3$-$^{12}COO^-$ | 4.4 | $^{13/12}C$ | |
| | $^{12}CH_3$-$^{13}COO^-$ | 8.6 | | |
| | $^{12}CH_3$-$^{12}COO^-$ | 7.4 | $^{12}C$ | |

**Table S4.** Detailed Summary of Ethanol Peaks from $^{1}H$ NMR Spectra (Figure S8). This table lists the isotopic composition, chemical shift, and integrated areas of each peak. Analysis was performed using the Multiplet Analysis in Mnova (version 15.0, Mestrelab).

| Strain | Specific isotope molecule | Peak's chemical shift (ppm) | Integrated Area (%) |
|---|---|---|---|
| | $^{13}CH_3$-$^{13}CH_2OH$ | 3.87 | 3.2 |
| | $^{12}CH_3$-$^{13}CH_2OH$ | 3.86 | 0.9[a] |
| | $^{13}CH_3$-$^{13}CH_2OH$ | 3.86 | 3.1 |
| | $^{13}CH_3$-$^{13}CH_2OH$ | 3.85 | 8.2 |
| | $^{12}CH_3$-$^{13}CH_2OH$ | 3.85 | 1.3 |
| | $^{13}CH_3$-$^{13}CH_2OH$ | 3.84 | 7.7 |
| | $^{13}CH_3$-$^{13}CH_2OH$ | 3.83 | 7.8 |
| | $^{12}CH_3$-$^{13}CH_2OH$ | 3.83 | 1.3 |
| | $^{13}CH_3$-$^{13}CH_2OH$ | 3.83 | 8.2 |
| | $^{13}CH_3$-$^{13}CH_2OH$ | 3.81 | 3.0 |
| | $^{12}CH_3$-$^{13}CH_2OH$ | 3.81 | 0.9[a] |
| | $^{13}CH_3$-$^{13}CH_2OH$ | 3.81 | 6.2 |
| ***Cl*adapt** | $^{13}CH_3$-$^{13}CH_2OH$ | 3.51 | 3.2 |
| | $^{12}CH_3$-$^{13}CH_2OH$ | 3.51 | 0.9[b] |
| | $^{13}CH_3$-$^{13}CH_2OH$ | 3.50 | 2.9 |
| | $^{13}CH_3$-$^{13}CH_2OH$ | 3.49 | 8.0 |
| | $^{12}CH_3$-$^{13}CH_2OH$ | 3.49 | 1.0 |
| | $^{13}CH_3$-$^{13}CH_2OH$ | 3.49 | 7.9 |
| | $^{13}CH_3$-$^{13}CH_2OH$ | 3.47 | 7.5 |
| | $^{12}CH_3$-$^{13}CH_2OH$ | 3.47 | 1.4 |
| | $^{13}CH_3$-$^{13}CH_2OH$ | 3.47 | 8.1 |
| | $^{13}CH_3$-$^{13}CH_2OH$ | 3.46 | 3.3 |
| | $^{12}CH_3$-$^{13}CH_2OH$ | 3.45 | 0.9 |
| | $^{13}CH_3$-$^{13}CH_2OH$ | 3.45 | 3.1 |

NB: Multiplet Analysis in Mnova was not able to peak fit $^{12}CH_3$-$^{13}CH_2OH$ peaks at 3.86 and 3.81 ppm in Figure S8A and 3.51 ppm in Figure S8B. [a] The integrated areas of $^{12}CH_3$-$^{13}CH_2OH$ peaks at 3.86 and 3.81 ppm in Figure S8A were estimated using the same relative integration area found between peaks at 3.45, 3.47 and 3.49 ppm in Figure S8B. [b] The same integrated area of $^{12}CH_3$-$^{13}CH_2OH$ peak at 3.45 ppm was used for 3.51 ppm, assuming both peaks have the same integrated area.

**Table S5.** Summary of Area Measurements for each ethanol isotope and the $^{13}C/^{12}C$ Ratio. This table presents a detailed analysis of the area contributions from different isotopes, along with the calculated $^{13}C/^{12}C$ ratios. For ethanol species $^{12}CH_3$-$^{13}CH_2OH$, which contains mixed $^{12}C$ and $^{13}C$ isotopes, the quantities were divided equally when calculating the $^{13}C/^{12}C$ ratio.

| Strain | Specific isotope molecule | Area (%) per isotope | Specific carbon isotopes | $^{13}C/^{12}C$ Ratio |
|---|---|---|---|---|
| ***Cl*adapt** | $^{12}CH_3$-$^{13}CH_2OH$ | 91.3 | $^{13}C$ | 96:4 |
| | $^{13}CH_3$-$^{13}CH_2OH$ | 8.7 | $^{13/12}C$ | |

**Table S6.** Mutations found in *Cl*adapt and *Cl*wt strains, relative to the NCBI reference sequence (NC_014328.1).

| Position and Mutation | REF | ALT | Gene | Sample |
|---|---|---|---|---|
| 85591 | C | T | *CLJU_RS00445* | *Cl*adapt |
| 883819 | G | A | *grpE* | *Cl*adapt |
| 1040146 | C | T | *CLJU_RS04595* | *Cl*adapt |
| 1430390 | G | GGCAGGAGCATTA | *CLJU_RS06530* | Shared |
| 1430479 | C | A | *CLJU_RS06530* | Shared |
| 1496011 | G | A | *CLJU_RS06845* | *Cl*adapt |
| 1614559 | G | A | *CLJU_RS07315* | *Cl*wt |
| 1614561 | C | T | *CLJU_RS07315* | *Cl*wt |
| 1619490 | CT | C | *rrf* | Shared |
| 1966227 | G | T | *deoC* | Shared |
| 2109343 | C | T | *CLJU_RS09515* | *Cl*adapt |
| 2235661 | T | G | *CLJU_RS10055* | Shared |
| 2533491 | C | T | *CLJU_RS11365* | *Cl*adapt |
| 2698277 | T | TA | *CLJU_RS22935* | Shared |
| 3079839 | T | C | non-coding | *Cl*adapt |
| 3352167 | T | C | *CLJU_RS14955* | *Cl*wt |
| 3352220 | C | T | *CLJU_RS14955* | *Cl*wt |
| 3352422 | T | C | *CLJU_RS14955* | *Cl*wt |
| 3352434 | C | T | *CLJU_RS14955* | *Cl*wt |
| 3352475 | C | T | *CLJU_RS14955* | *Cl*wt |
| 3352548 | C | T | *CLJU_RS14955* | *Cl*wt |
| 3352599 | C | T | *CLJU_RS14955* | Shared |
| 3352602 | T | C | *CLJU_RS14955* | Shared |
| 3352611 | C | T | *CLJU_RS14955* | Shared |
| 3352677 | C | T | *CLJU_RS14955* | Shared |
| 3352736 | G | A | *CLJU_RS14955* | Shared |
| 3352780 | T | G | *CLJU_RS14955* | Shared |
| 3352838 | A | C | *CLJU_RS14955* | *Cl*wt |
| 3352950 | T | C | *CLJU_RS14955* | *Cl*wt |
| 3352953 | T | C | *CLJU_RS14955* | *Cl*wt |
| 3353157 | T | A | *CLJU_RS14955* | *Cl*wt |
| 3353159 | G | C | *CLJU_RS14955* | *Cl*wt |
| 3353160 | T | A | *CLJU_RS14955* | *Cl*wt |
| 3353163 | T | C | *CLJU_RS14955* | *Cl*wt |
| 3353230 | A | G | *CLJU_RS14955* | Shared |
| 3353348 | C | A | *CLJU_RS14955* | *Cl*wt |
| 3353396 | C | T | *CLJU_RS14955* | Shared |
| 3353651 | T | C | *CLJU_RS14955* | *Cl*wt |

| | | | | |
|---|---|---|---|---|
| 3353655 | A | G | *CLJU_RS14955* | Shared |
| 3867526 | C | A | non-coding | *Cl*adapt |
| 4428830 | A | C | *CLJU_RS20060* | Shared |

**Table S7.** Summary of Functional Mutations in Genes Differentiating the Adapted (*Cl*adapt) and Wild-Type (*Cl*wt) Strains of *Clostridium ljungdahlii*.

| Strain | Position and Mutation | Amino Acid Changes | Gene | Gene product functions[a] |
|---|---|---|---|---|
| *Cl*adapt | 85591, C → T | Ala → Val | *CLJU_RS00445* | Deoxyribose-phosphate aldolase |
| | 883819, G → A | Glu → Lys | *grpE* | Nucleotide exchange factor |
| | 1040146, C → T | silent mutations | *CLJU_RS04595* | Sugar-specific transcriptional regulator |
| | 1496011, G → A | Gly → Asp | *CLJU_RS06845* | Oligopeptide transporter |
| | 2109343, C → T | silent mutations | *CLJU_RS09515* | Phenazine biosynthesis |
| | 2533491, C → T | Thr → Ile | *CLJU_RS11365* | Nitrogenase molybdenum iron protein |
| | 3079839, T → C | - | *non-coding* | unknown |
| | 3867526, C → A | - | *non-coding* | unknown |
| *Cl*wt | 1614559, G → A | - | *CLJU_RS07315* | 16S ribosomal RNA |
| | 1614561, C → T | - | | |
| | 3352167, T → C | silent mutations | *CLJU_RS14955* | LTD domain-containing protein, function unknown |
| | 3352220, C → T | Asp → Asn | | |
| | 3352422, T → C | silent mutations | | |
| | 3352434, C → T | silent mutations | | |
| | 3352475, C → T | Asp → Asn | | |
| | 3352548, C → T | silent mutations | | |
| | 3352838, A → C | Ser → Ala | | |
| | 3352950, T → C | silent mutations | | |
| | 3352953, T → C | silent mutations | | |
| | 3353157, T → A | Pro → Ala | | |
| | 3353159, G → C | Pro → Ala | | |
| | 3353160, T → A | Lys → Asn | | |
| | 3353163, T → C | silent mutations | | |
| | 3353348, C → A | Ala → Ser | | |
| | 3353651, T→ C | Ile → Val | | |

[a] The functions of the gene products were inferred by analyzing structural similarities in the AlphaFold Protein Structure Database, with clustering performed using Foldseek Cluster.[8]

**Table S8**. Summary of reported photocatalytic flow systems for $CO_2$ reduction.

| Co-catalyst (μM) | Photocatalyst (mg)[a] | Sacrificial donor (M) | $CO_2$ flow rate (ml $min^{-1}$) | $CO_2$-derived products | Activity (mmol $g^{-1}$ $min^{-1}$) | Accumulated product (mmol) / time (h) | Yield (mmol $g_{photocatalyst}^{-1}$) | Product selectivity (%) | Ref. |
|---|---|---|---|---|---|---|---|---|---|
| Co(tppS3N1) (10) | ZnSe-$BF_4$ (0.5 μM)[a] | Ascorbic acid (0.1) | 4 | CO | 0.1 | 18.6 x $10^{-3}$ (17) | 79.7 | 41 | [9] |
| $Cu_2O$ | Reduced $TiO_2$ (40) | $H_2O$ | 1.2 | $CH_4$ | 1.3 x $10^{-6}$ | 18.5 x $10^{-6}$ (6) | 462 x $10^{-6}$ | | [10] |
| Pt | $TiO_2$ P25 on a GDE (25) | $H_2O$ | 10 | CO and $CH_4$ | 5.4 and 0.4 x $10^{-3}$ | 0.10 (12) | 3.9 and 0.3 | 93, 7 | [11] |
| - | $TiO_2$ P25 (70) | $H_2O$ | 5 | $CH_4$ | 5 x $10^{-6}$ | 1.4 x $10^{-4}$ (6.8) | 2.0 x $10^{-3}$ | 100 | [12] |
| - | $Bi_2WO_6$ (15) | $H_2O$ | 0.4 | CO | 1.3 x $10^{-5}$ | 1.2 x $10^{-2}$ (4) | 3.1 x $10^{-3}$ | 99 | [13] |
| Cu | $TiO_2$ | $H_2O$ | 3 | $CH_3OH$ | 6.9 x $10^{-6}$ | - | 4.2 x $10^{-4}$ | 100 | [14] |
| Cu-Fe | $TiO_2$ P25 | $H_2O$ | 5 | $C_2H_4$ and $CH_4$ | 9.7 and 15.2 x $10^{-6}$ | - | 2.3 and 3.6 x $10^{-3}$ | 39, 61 | [15] |
| NiO | $InTaO_4$ (125) | $H_2O$ | | $CH_3OH$ and acetaldehyde | 2.7 and 5.0 x $10^{-6}$ | 1.2 and 2.3 x $10^{-4}$ (6) | 9.6 and 18.0 x $10^{-7}$ | 35, 65 | [16] |
| CotpyP (7.3) | $TiO_2$ P25 (365) | TEOA (0.1) | 30 | CO | 1.5 x $10^{-6}$ | 1.3 (144) | 3.5 | 29 | This work |

[a] Unit is milligram, unless otherwise stated.

**Table S9.** Summary of photocatalytic syngas generation by $TiO_2$|CotpyP hybrid in $CO_2$-saturated 0.1 M TEOA aqueous solutions under batch and flow modes.

| Experiment mode | $Yield_{CO}$ / µmol CO $g_{TiO2}^{-1}$ | $Yield_{H2}$ / µmol $H_2$ $g_{TiO2}^{-1}$ | $TON_{CO}$ |
|---|---|---|---|
| Batch[a] (before bioreactor) | 899 ± 377 | 3286 ± 1056 | 43 ± 18 |
| Batch[a] (after bioreactor) | 602 ± 292 | 869 ± 1421[d] | 29 ± 14 |
| Experiment mode | Activity CO / nmol CO $g_{TiO2}^{-1}$ $min^{-1}$ | Activity $H_2$ / nmol $H_2$ $g_{TiO2}^{-1}$ $min^{-1}$ | |
| Flow[b] | 148 ± 97 | 367 ± 606[e] | |
| | n. d.[c] | n. d.[c] | |

[a] Batch experiments = 24 h (see **Figure S15**); [b] in all flow mode experiments, $CO_2$ containing 2% $CH_4$ was continuously flowed at 30 mL $min^{-1}$; [c] control experiment without light. Turnover number (TON) is calculated by dividing mol of gaseous product by mol of catalyst. [d] The average of $H_2$ yield obtained from batch experiments after six days of connection to a bioreactor (869.4 ± 1420.8 µmol $H_2$ $g_{TiO2}^{-1}$) was lower than its standard deviation because $H_2$ was completely consumed in two of the experiments, while in a third experiment, a significant amount of $H_2$ remained. This led to an average value lower than its standard deviation. [e] The average rate values are lower than their standard deviation due to variations in the measured $H_2$ gas production. Specifically, one of the experiments generated significantly more $H_2$ than the others, resulting in a lower average value and a much larger standard deviation (367 ± 606 nmol $H_2$ $g_{TiO2}^{-1}$ $min^{-1}$).

***References***


1 J. M. A. Al-Rawi, J. A. Elvidge, J. R. Jones and E. A. Evans, *J. Chem. Soc., Perkin Trans. 2*, 1975, **0**, 449–452.

2 B. W. Koenig and K. Gawrisch, *J. Phys. Chem. B*, 2005, **109**, 7540–7547.

3 H. Liu, J. Zhang, J. Yuan, X. Jiang, L. Jiang, G. Zhao, D. Huang and B. Liu, *Biotechnol. Biofuels*, 2019, **12**, 101.

4 J. P. Petzel, M. C. McElwain, D. DeSantis, J. Manolukas, M. V. Williams, P. A. Hartman, M. J. Allison and J. D. Pollack, *Arch. Microbiol.*, 1989, **152**, 309–316.

5 C. Harrison, *Cell Stress Chaperones*, 2003, **8**, 218–224.

6 R. Gardan, C. Besset, A. Guillot, C. Gitton and V. Monnet, *J. Bacteriol.*, 2009, **191**, 4647–4655.

7 M. S. Weininger and L. E. Mortenson, *Proc. Natl. Acad. Sci.*, 1982, **79**, 378–380.

8 I. Barrio-Hernandez, J. Yeo, J. Jänes, M. Mirdita, C. L. M. Gilchrist, T. Wein, M. Varadi, S. Velankar, P. Beltrao and M. Steinegger, *Nature*, 2023, **622**, 637–645.

9 C. D. Sahm, G. M. Ucoski, S. Roy and E. Reisner, *ACS Catal.*, 2021, **11**, 11266–11277.

10 S. Ali, J. Lee, H. Kim, Y. Hwang, A. Razzaq, J.-W. Jung, C.-H. Cho and S.-I. In, *Appl. Catal. B: Environ.*, 2020, **279**, 119344.

11 H. Jung, C. Kim, H.-W. Yoo, J. You, J. S. Kim, A. Jamal, I. Gereige, J. W. Ager and H.-T. Jung, *Energy Environ. Sci.*, 2023, **16**, 2869–2878.

12 M. Dilla, R. Schlögl and J. Strunk, *ChemCatChem*, 2017, **9**, 696–704.

13 C. S. Ribeiro, J. Z. Y. Tan, M. M. Maroto-Valer and M. A. Lansarin, *J. Environ. Chem. Eng.*, 2021, **9**, 105097.

14 J. C. S. Wu, H.-M. Lin and C.-L. Lai, *Appl. Catal. A: Gen.*, 2005, **296**, 194–200.

15 T.-V. Nguyen and J. C. S. Wu, *Appl. Catal. A: Gen.*, 2008, **335**, 112–120.

16 P.-Y. Liou, S.-C. Chen, J. C. S. Wu, D. Liu, S. Mackintosh, M. Maroto-Valer and R. Linforth, *Energy Environ. Sci.*, 2011, **4**, 1487–1494.


End of ESI
